# Surrogate endpoints in mature B-cell neoplasms – meaningful or misleading?

**DOI:** 10.1038/s41375-024-02483-5

**Published:** 2024-12-10

**Authors:** Florian Simon, Othman Al-Sawaf, John F. Seymour, Barbara Eichhorst

**Affiliations:** 1 Faculty of Medicine and University Hospital Cologne, Department I of Internal Medicine, Center for Integrated Oncology Aachen Bonn Cologne Duesseldorf, University of Cologne, Cologne, Germany; 2 Department of Haematology Peter MacCallum Cancer Centre & Royal Melbourne Hospital, University of Melbourne, Melbourne, VIC Australia

**Keywords:** B-cell lymphoma, Cancer epidemiology

## Abstract

Indolent mature B-cell neoplasms are a group of diseases in which recent therapeutic advances have led to an improved overall survival (OS) extending beyond several years. While cause of celebration for patients and caregivers, the increasingly long observation periods necessary to capture treatment effects are complicating trial design and possibly hindering swift access to more effective therapies. Surrogate endpoints are a tool with the potential of earlier study readouts, however, their validity needs to be proven in each individual disease and therapeutic setting. The validation of surrogate endpoints and available data for mature B-cell neoplasms are discussed within this perspective article, followed by an outlook on the potential of precise tools such as measurable residual disease assessment as novel surrogate candidates.

## Introduction

The overarching aim of treating cancer is optimally cure. The term cancer cure refers to an enduring complete clinical remission of a cancer, regardless of the presence or absence of late sequelae of treatments, for the duration of the patient’s natural life span [1]. In order to measure potential cure according to that definition, overall survival (OS) is considered the most important endpoint in clinical trials. However, OS has shifted from being the primary to secondary endpoint in most clinical trials in hematologic malignancies due to the often very long observation periods being required [2]. As an example, the recent advances in the treatment of indolent lymphoma result in very long survival times for most patients, reaching the normal life expectancy in some subgroups. Hence, OS as the primary endpoint is practically difficult to measure within the timeframe of an individual trial in this disease entity because follow-up times of 10 years or longer would be required. Besides OS, health related quality of live (HRQOL) plays an important role as an outcome parameter in indolent lymphoma, noting this may also be affected by patients’ comorbidities with increasing age. These aspects underline the importance of defining surrogate endpoints as helpful tools to provide early estimates of expected improvements in OS.

Though there are many examples of clinical trials demonstrating that - in particular during frontline therapy - prolongation of progression-free survival (PFS) resulted in a prolongation of OS [3–5], longer PFS is not always a reliable adequate surrogate parameter for OS [6, 7], partly due to the increasing number and efficacy of options for salvage treatments.

Additionally, being a victim of its own success, even PFS differences are becoming increasingly difficult to capture in indolent lymphoma and therefore the use of an earlier and possibly even more precise measure of treatment success is enticing to trialists as well as pharmaceutical companies: measurable or minimal residual disease (MRD) measured from peripheral blood or bone marrow has all the theoretical and biological plausibility to fulfill just this promise: it distinguishes up to a level of 1 in 10.000 cells the (remaining) presence of a malignant cell and has a higher precision than imaging response in indolent lymphoma [8, 9]. Lower levels of MRD should therefore inevitably lead to longer time to progression, at least on a trial/patient cohort level.

Although the prognostic impact of MRD on survival has been well established in CLL [10] and FL [11] and its use as an intermediate endpoint for clinical trials has been accepted by FDA and EMA [12], the data on its use a surrogate endpoint is scant within this context. Interestingly, on April 12th of 2024 the oncologic drugs advisory committee (ODAC) of the FDA has voted for the use of minimal residual disease as a surrogate endpoint for accelerated approval of new treatment for multiple myeloma [13].

In this perspective response parameters such as MRD, but also complete response and overall response as well as PFS as surrogate endpoints in indolent lymphoproliferative disorders are discussed with respect to their validation, potential use and limitations.

## Surrogate endpoints and their validation

A surrogate endpoint as accepted by European health care stakeholders (European network for health technology assessment (EUnetHTA)) [14] and the federal drug administration (FDA) [15] is an intermediate endpoint which is not only prognostic for the true endpoint but also captures the extent to which a treatment influences the true endpoint.

To formally validate surrogate endpoints different statistical approaches have been proposed, which mostly rely on a two-level meta-analytic method [16, 17], in which both patient and trial level correlation of surrogate endpoints are calculated, ideally based on individual patient data.

**Fig. 1:**
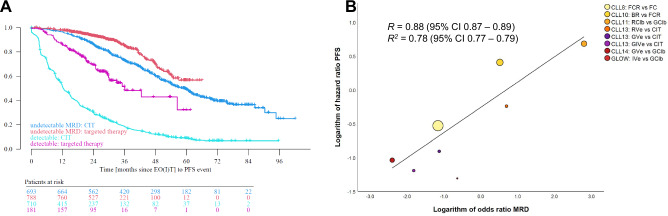
Examples of validation for surrogate endpoints. **A** End of treatment MRD status shows a correlation with overall survival on a patient-level, i.e. it has significant prognostic value. **B** The odds ratio of reaching undetectable MRD across multiple trials is strongly associated with the hazard ratios of PFS, i.e. trial-level correlation. Figure adapted from End Point Surrogacy in First-Line Chronic Lymphocytic Leukemia. Florian Simon et al., JCO 0, JCO.24.01192 [22]. Printed with permission.

Patient level correlation describes the degree with which a surrogate and the true endpoint are associated on an individual patient level or more specifically the prognostic value of the endpoint (Fig. 1). Trial level correlation is however vital to validate a surrogate endpoint and its use in the setting of clinical trials: It reflects the association of treatment effects on both endpoints, e.g. the correlation of the hazard ratio (HR) of PFS with the HR of OS in a randomized trial. Ideally, if validated through a meta-analytic approach, the effect on OS can be estimated through the impact on the surrogate or surrogate threshold effects [18] can be determined, i.e. the threshold HR PFS from which an HR OS difference can be assumed.

However, there is still no formal international consensus on the optimal statistical method with which both effects should be measured, and thresholds remain arbitrarily defined. While some statisticians argue that any value above 0.5 for correlation coefficients between surrogate and true endpoints might suffice, recent publications have set thresholds above >0.8 for strong and <0.6 as weak correlations [19].

Additionally, limitations in the application of the two-level approach remain, as they do not account for differential censoring of data and rely on a large number of trials/trial comparisons [20] and especially events to precisely define treatment effects, an increasingly difficult requirement in the setting of indolent lymphomas.

## Surrogate endpoints in mature B-cell neoplasms

In CLL, the most commonly used surrogate endpoints in phase 2 and 3 clinical trials are overall and complete response rates, rates of undetectable measurable residual disease and PFS (Table 1). Within a set of chemotherapy trials, a high patient-level correlation of 0.8 was shown between PFS and OS [21]. Similar observations were made with chemo/chemoimmuntherapy and limited-duration targeted therapy with patient-level correlation of >0.8 [22]. Trial-level surrogacy between PFS and OS with targeted therapies, including continuous BTK inhibition, was >0.7, indicating a moderate correlation between PFS and OS.

**Table 1:** Available data on surrogate endpoints in mature B-cell neoplasms.

Authors/Study	Lymphoma Subtype	Numbers of patients/trials	IPD	Surrogate Endpoint/True Endpoint	Individual /patient- level surrogacy	Trial-level surrogacy
Beauchemin et al. [21]	CLL	23 trials	No	PFS/OS	Rho: 0.813	NA
Shi et al. [23]	FL	13 trials, 3837 patients	Yes	OR CR30/HR PFS	OR CR30/PFS 11.8	*R*^2^_WLS_: 0.88 *R*^2^_Copula_:0.86
Zhu et al. [33]	FL MCL	52 trials, 5542 patients 32 trials, 1425 patients	No	6MoPFS/3year PFS 6MoPFS/Median PFS 6MoPFS/2year OS 6MoPFS/Median OS	R^2^: 0.89 R^2^: 0.66 R^2^: 0.69 R^2^: 0.71	NA
Simon et al. [22]	CLL	12 trials, 4237 patients + external dataset 9 trials with 3993 patients	Yes + No for external dataset	PFS/OS MRD/PFS MRD/OS	24MoPFS/60Mo OS: Rho 0.97 HR PFS uMRD vs. MRD: 4.74 HR OS uMRD vs. MRD: 3.02	HR PFS/OS *R*: 0.75 *R*^2^: 0.56 OR MRD/HR PFS *R*: 0.88 *R*^2^: 0.78 OR MRD/HR OS *R*: 0.71 *R*^2^: 0.5
Bahar et al. [29]	CLL	29 trials	No	ORR/PFS PFS/OS	Rho = 0.58	Rho = 0.58
Milrod et al. [34]	FL	22 trials, 10,729 patients	No	HR PFS/OS	NA	LWR: 0.383 *R*^2^: 0.15

*CLL* chronic lymphocytic leukemia, *FL* follicular lymphoma, *IPD* individual patient data, *WLS* weighted least squares, *LWR* linear weighted regression, *HR* Hazard ratio, *OR* Odds ratio, *PFS* Progression-free survival, *OS* Overall survival, *MRD* measurable residual disease.

In the context of follicular lymphoma (FL) the best validated trial-level surrogate for long-term PFS is the proportion of patients remaining in ongoing CR at 30 months after initiation of their frontline therapy (CR30) [23]. It was prospectively identified by the global Follicular Lymphoma Analysis of Surrogate Hypothesis (FLASH) consortium from their individual patient data (IPD)-based analysis of 13 chemo-immunotherapy trials with 3837 patients with a strong correlation of *R*^2^ of 0.88. CR30 was also validated as a robust surrogate for PFS at an individual patient level and had superior performance to CR24. In the analysis by Shi et al., a threshold of 11% absolute improvement in CR30 from a 50% control rate, predicted a significant treatment effect on PFS, demonstrating its applicability as surrogate endpoint for PFS [23]. Although, CR30 has not been examined as a predictor for OS in FL, it is currently implemented as a co-primary endpoint in several ongoing trials in frontline FL. On the other hand, although widely accepted as a strong predictor of adverse outcome, POD24 did not perform well as a surrogate for OS in either FL [24] or MZL [25].

In advanced stage marginal zone lymphoma (MZL), CR24 (as distinct from CR30) has been explored as a potential surrogate for PFS using IPD from the 401 patients in the IELSG19 3 arm study and found to be a robust surrogate for 8-year PFS rate [26].

While the US FDA policies state that they are supportive of considering regulatory submissions in lymphoma based on “durable response” rates and some recent approvals of the covalent BTK inhibitors ibrutinib and zanubrutinib in Waldenstrom macroglobulinemia have cited this criterion, the exact definition of “durable” and what proportion of responses are required to show such durability are unclear, and the formal surrogacy of this endpoint has not been established [27].

## MRD as a surrogate endpoint

### CLL

In CLL, response endpoints like undetectable MRD rates were strongly associated with PFS on a trial level (*R* > 0.8), thereby suggesting its utility as an intermediate endpoint. However, the correlation between MRD and OS was modest with an *R* of 0.71 but limited by a small number of datapoints for these events. Definitive conclusions on MRD surrogacy for OS can therefore not yet be concluded [28]. Conversely, ORR does not correlate with OS across different treatment modalities [29]. Overall, the available evidence supports the use of time-to-event endpoints as primary outcome measures in randomized CLL studies, while use of response endpoints requires further surrogacy validation.

### Indolent lymphoma

While optimal methodology and international harmonization of MRD assays in indolent NHL lag far behind CLL, these do have some emerging promise as potential surrogate endpoint in follicular NHL [11] based on an analysis of long-term outcome in the GALLIUM study and using PCR of t(14;18) translocation and/or clonal Ig rearrangement at a 10^−5^ level of sensitivity, showing an overall strong association with PFS, but with differential impacts seen across different treatment regimens (NOT prognostic in obinutuzumab-treated patients), underlining that further evaluation is necessary before this could be generalized. In that same study, PET response status at end-of-induction therapy added to the prognostic performance of MRD status, supporting further exploration of PET response parameters as another potential surrogate.

## Conclusion

Especially in mature B-cell neoplasms OS difference is increasingly difficult to capture within the practical timeframe of clinical trials. For example, CLL14 has shown clear superiority of venetoclax + obinutuzumab vs. chlorambucil + obinutuzumab in terms of PFS but OS differences have so far not crossed significance boundaries even after 6 years of follow-up [30].

When trying to validate endpoints, even with large datasets, correlation of surrogate candidates such as PFS, Time to next treatment (TTNT), response rates (CR30) or MRD with OS is difficult to evaluate due to few events as well as the efficacy of relapse treatments, especially in the setting of novel agents. The recent unanimous ODAC-vote on the use of MRD as a regulatory endpoint in multiple myeloma based on a similar amount of data to that available in CLL however, shows the trust which trialists put into this promising response assessment. Additionally, novel approaches to assess MRD with a higher resolution such as NGS-based/CAPP-sequencing approaches, which showed higher prognostic value [31, 32], might eventually lead to the granularity needed to validate MRD as a true surrogate endpoint for OS.

Finally, even if analyses ultimately show that there is only a moderate or weak correlation, PFS or MRD and their impact on patients’ quality of life should not be underestimated, especially when a long-lasting remission is achieved through a well-tolerated time-limited treatment.

Combined efforts across the scientific community are required to enable the analysis of large, aggregated datasets to strengthen the significance of surrogate parameters.

## References

[1] Tralongo P, Maso LD, Surbone A, Santoro A, Tirelli U, Sacchini V, et al. Use of the word “cured” for cancer patients—implications for patients and physicians: the Siracusa charter. Curr Oncol. 2015;22:38–40.

[2] Royle K-L, Meads D, Visser-Rogers JK, White IR, Cairns DA. How is overall survival assessed in randomised clinical trials in cancer and are subsequent treatment lines considered? A systematic review. Trials. 2023;24:708. 37926806 10.1186/s13063-023-07730-1 PMC10626781

[3] Shanafelt TD, Wang XV, Kay NE, Hanson CA, O’Brien S, Barrientos J, et al. Ibrutinib–rituximab or chemoimmunotherapy for chronic lymphocytic leukemia. N Engl J Med. 2019;381:432–43. 31365801 10.1056/NEJMoa1817073 PMC6908306

[4] Munir T, Cairns DA, Bloor A, Allsup D, Cwynarski K, Pettitt A, et al. Chronic lymphocytic leukemia therapy guided by measurable residual disease. N Engl J Med. 2024;390:326–37. 38078508 10.1056/NEJMoa2310063

[5] Hiddemann W, Kneba M, Dreyling M, Schmitz N, Lengfelder E, Schmits R, et al. Frontline therapy with rituximab added to the combination of cyclophosphamide, doxorubicin, vincristine, and prednisone (CHOP) significantly improves the outcome for patients with advanced-stage follicular lymphoma compared with therapy with CHOP alone: results of a prospective randomized study of the German Low-Grade Lymphoma Study Group. Blood. 2005;106:3725–32. 16123223 10.1182/blood-2005-01-0016

[6] Hillmen P, Pitchford A, Bloor A, Broom A, Young M, Kennedy B, et al. Ibrutinib and rituximab versus fludarabine, cyclophosphamide, and rituximab for patients with previously untreated chronic lymphocytic leukaemia (FLAIR): interim analysis of a multicentre, open-label, randomised, phase 3 trial. Lancet Oncol. 2023;24:535–52. 37142374 10.1016/S1470-2045(23)00144-4

[7] Marcus R, Davies A, Ando K, Klapper W, Opat S, Owen C, et al. Obinutuzumab for the first-line treatment of follicular lymphoma. N Engl J Med. 2017;377:1331–44. 28976863 10.1056/NEJMoa1614598

[8] Al-Sawaf O, Zhang C, Tandon M, Sinha A, Fink A-M, Robrecht S, et al. Venetoclax plus obinutuzumab versus chlorambucil plus obinutuzumab for previously untreated chronic lymphocytic leukaemia (CLL14): follow-up results from a multicentre, open-label, randomised, phase 3 trial. Lancet Oncol. 2020;21:1188–200. 32888452 10.1016/S1470-2045(20)30443-5

[9] Dimier N, Delmar P, Ward C, Morariu-Zamfir R, Fingerle-Rowson G, Bahlo J, et al. A model for predicting effect of treatment on progression-free survival using MRD as a surrogate end point in CLL. Blood. 2018;131:955–62. 29255066 10.1182/blood-2017-06-792333

[10] Wierda WG, Rawstron A, Cymbalista F, Badoux X, Rossi D, Brown JR, et al. Measurable residual disease in chronic lymphocytic leukemia: expert review and consensus recommendations. Leukemia. 2021;35:3059–72. 34168283 10.1038/s41375-021-01241-1 PMC8550962

[11] Pott C, Jurinovic V, Trotman J, Kehden B, Unterhalt M, Herold M, et al. Minimal residual disease status predicts outcome in patients with previously untreated follicular lymphoma: a prospective analysis of the phase III GALLIUM study. J Clin Oncol. 2024;42:550–61. 38096461 10.1200/JCO.23.00838

[12] Committee for Medicinal Products for Human Use (CHMP). Appendix 4 to the guideline on the evaluation of anticancer medicinal products in man 2016 [Available from: https://www.ema.europa.eu/en/documents/scientific-guideline/evaluation-anticancer-medicinal-products-man-appendix-4-condition-specific-guidance-revision-2_en.pdf.

[13] FDA’s ODAC Recognizes MRD as an Accepted End Point for Accelerated Approval in Multiple Myeloma [Available from: https://www.onclive.com/view/fda-s-odac-recognizes-mrd-as-an-accepted-end-point-for-accelerated-approval-in-multiple-myeloma.

[14] Endpoints used in Relative Effectiveness Assessment: Surrogate Endpoints. https://www.eunethta.eu/wp-content/uploads/2018/01/Endpoints-used-in-Relative-Effectiveness-Assessment-Surrogate-Endpoints_Amended-JA1-Guideline_Final-Nov-2015.pdf2015.

[15] US Department of Health and Human Services. Food and Drug Administration. Clinical trial endpoints for the approval of cancer drugs and biologics: Guidance for Industry 2018 [Available from: https://www.fda.gov/regulatory-information/search-fda-guidance-documents/clinical-trial-endpoints-approval-cancer-drugs-and-biologics.

[16] Buyse M, Molenberghs G, Burzykowski T, Renard D, Geys H. The validation of surrogate endpoints in meta-analyses of randomized experiments. Biostatistics. 2000;1:49–67. 12933525 10.1093/biostatistics/1.1.49

[17] Burzykowski T, Molenberghs G, Buyse M, Geys H, Renard D. Validation of surrogate end points in multiple randomized clinical trials with failure time end points. J R Stat Soc Ser C Appl Stat. 2002;50:405–22.

[18] Burzykowski T, Buyse M. Surrogate threshold effect: an alternative measure for meta-analytic surrogate endpoint validation. Pharm Stat. 2006;5:173–86. 17080751 10.1002/pst.207

[19] Shi Q, Schmitz N, Ou FS, Dixon JG, Cunningham D, Pfreundschuh M, et al. Progression-free survival as a surrogate end point for overall survival in first-line diffuse large B-cell lymphoma: an individual patient-level analysis of multiple randomized trials (SEAL). J Clin Oncol. 2018;36:2593–602. 29975624 10.1200/JCO.2018.77.9124 PMC6532366

[20] Sofeu CL, Emura T, Rondeau V. A joint frailty-copula model for meta-analytic validation of failure time surrogate endpoints in clinical trials. Biom J. 2021;63:423–46. 33006170 10.1002/bimj.201900306

[21] Beauchemin C, Johnston JB, Lapierre M, Aissa F, Lachaine J. Relationship between progression-free survival and overall survival in chronic lymphocytic leukemia: a literature-based analysis. Curr Oncol. 2015;22:e148–56. 26089725 10.3747/co.22.2119 PMC4462536

[22] Simon F, Ligtvoet R, Robrecht S, Cramer P, Kutsch N, Fürstenau M, et al. Endpoint surrogacy in first-line chronic lymphocytic leukemia. J Clin Oncol. 10.1200/JCO.24.01192. 10.1200/JCO.24.01192 PMC11771364 39213466

[23] Shi Q, Flowers CR, Hiddemann W, Marcus R, Herold M, Hagenbeek A, et al. Thirty-month complete response as a surrogate end point in first-line follicular lymphoma therapy: an individual patient-level analysis of multiple randomized trials. J Clin Oncol. 2017;35:552–60. 28029309 10.1200/JCO.2016.70.8651

[24] Bachy E, Cerhan JR, Salles G. Early progression of disease in follicular lymphoma is a robust correlate but not a surrogate for overall survival. Blood Adv. 2021;5:1729–32. 33729455 10.1182/bloodadvances.2020003797 PMC7993099

[25] Luminari S, Merli M, Rattotti S, Tarantino V, Marcheselli L, Cavallo F, et al. Early progression as a predictor of survival in marginal zone lymphomas: an analysis from the FIL-NF10 study. Blood. 2019;134:798–801. 31292118 10.1182/blood.2019001088

[26] Bommier C, Zucca E, Chevret S, Conconi A, Nowakowski G, Maurer MJ, et al. Early complete response as a validated surrogate marker in extranodal marginal zone lymphoma systemic therapy. Blood. 2024;143:422–8.37801707 10.1182/blood.2023020984

[27] Walia A, Haslam A, Prasad V. FDA validation of surrogate endpoints in oncology: 2005–2022. J Cancer Policy. 2022;34:100364. 36155118 10.1016/j.jcpo.2022.100364

[28] Simon F, Ligtvoet R, Robrecht S, Cramer P, Kutsch N, Furstenau M, et al. Endpoint surrogacy in chronic lymphocytic leukemia: a pooled analysis of the German CLL study group. Blood. 2023;142:1901.

[29] Bahar N, Mohseninejad L, McDonald K, Wilke T. A meta-analytic endpoint validation of surrogates used in clinical trials evaluating the efficacy of therapies in patients with chronic lymphocytic leukemia (CLL). Hematol Oncol. 2023;41:743–4.37086447

[30] Al-Sawaf O, Robrecht S, Zhang C, Olivieri S, Chang YM, Fink AM, et al. Venetoclax-Obinutuzumab for previously untreated chronic lymphocytic leukemia: 6-year results of the phase 3 CLL14 study. Blood. 2024;144:1924–1935. 10.1182/blood.2024024631 PMC11551846 39082668

[31] Hengeveld PJ, van der Klift MY, Kolijn PM, Davi F, Kavelaars FG, de Jonge E, et al. Detecting measurable residual disease beyond 10-4 by an IGHV leader-based NGS approach improves prognostic stratification in CLL. Blood. 2023;141:519–28. 36084320 10.1182/blood.2022017411

[32] Scherer F, Kurtz DM, Newman AM, Stehr H, Craig AF, Esfahani MS, et al. Distinct biological subtypes and patterns of genome evolution in lymphoma revealed by circulating tumor DNA. Sci Transl Med. 2016;8:364ra155. 27831904 10.1126/scitranslmed.aai8545 PMC5490494

[33] Zhu R, Lu D, Chu YW, Chai A, Green M, Zhang N, et al. Assessment of Correlation Between Early and Late Efficacy Endpoints to Identify Potential Surrogacy Relationships in Non-Hodgkin Lymphoma: a Literature-Based Meta-analysis of 108 Phase II and Phase III Studies. AAPS J. 2017;19:669–81. 28224402 10.1208/s12248-017-0056-x

[34] Milrod CJ, Kim KW, Raker C, Ollila TA, Olszewski AJ, Pelcovits A Progression-free survival is a weakly predictive surrogate end-point for overall survival in follicular lymphoma: a systematic review and meta-analysis. Br J Haematol. 2024;204:2237–2241. 10.1111/bjh.19449 38571449

